# Breast Cancer-Associated Adipose Tissue Histologic Subtypes: Microscopic Characterization and Their Impact on Prognosis and Survival, Depending on Age

**DOI:** 10.3390/cancers18060966

**Published:** 2026-03-17

**Authors:** Mihaela Maria Pasca Fenesan, Razvan George Bogdan, Andrei Alexandru Cosma, Vlad Vornicu, Eugen Melnic, Diana Veronica Radu, Patricia Baran, Zorin Crainiceanu, Ana Silvia Corlan, Anca Maria Cimpean, Peter Seropian, Olga Cernetchi, Ionut Marcel Cobec

**Affiliations:** 1 Doctoral School, “Victor Babes” University of Medicine and Pharmacy, 300041 Timisoara, Romania; mihaela.fenesan@umft.ro (M.M.P.F.); razvan.bogdan@umft.ro (R.G.B.); vlad.vornicu@umft.ro (V.V.);; 2 Oncohelp Clinic, 300425 Timisoara, Romania; cosma.andrei@umft.ro; 3 Department of Microscopic Morphology/Histology, “Victor Babes” University of Medicine and Pharmacy, 300041 Timisoara, Romania; 4 Plastic Surgery Department, “Victor Babes” University of Medicine and Pharmacy, 300041 Timisoara, Romania; crainiceanu.zorin@umft.ro; 5 Department of Pathology, Nicolae Testemitanu State University of Medicine and Pharmacy, 2300 Chisinau, Moldova; 6 Food Technologies Department, University of Life Sciences King Mihai I, 300645 Timisoara, Romania; dianadogaru@usvt.ro; 7 Department of Internal Medicine II, Discipline of Endocrinology, “Victor Babeș” University of Medicine and Pharmacy, 300041 Timisoara, Romania; ana.corlan@umft.ro; 8 Center of Expertise for Rare Vascular Disease in Children, Emergency Hospital for Children Louis Turcanu, 300011 Timisoara, Romania; 9 Center of Genomic Medicine, “Victor Babes” University of Medicine and Pharmacy, 300041 Timisoara, Romania; 10 Research Center for Pharmaco-Toxicological Evaluation, Victor Babes University of Medicine and Pharmacy, 300041 Timisoara, Romania; 11 Clinic of Obstetrics and Gynecology, Klinikum Freudenstadt, 72250 Freudenstadt, Germany; 12 Department of Obstetrics and Gynecology, Nicolae Testemitanu State University of Medicine and Pharmacy, 2300 Chisinau, Moldova; olga.cernetchi@usmf.md; 13 Department of Obstetrics and Gynecology, Faculty of Medicine, Medical Center-University of Freiburg, 79106 Freiburg, Germany

**Keywords:** breast cancer-associated adipose tissue (BCAAT), survival, invasion, prognosis

## Abstract

**Background/Objectives**: The fundamental classification based on white, brown, pink, and beige adipose tissue morphology together with fat vacuole content released into the tumor microenvironment incompletely defines breast cancer-associated adipose tissue (BCAAT) heterogeneity and does not sufficiently explain its controversial impact on invasion, recurrence, or survival in breast cancer (BC). We aim to expand BCAAT characterization by systematically evaluating stromal cellular elements within peritumoral adipose tissue, including CD34-positive fibroblasts, smooth muscle actin (SMA)-positive myofibroblasts, inflammatory cells, and microvascular structures to define distinct BCAAT subgroups. **Methods**: CD34 and smooth muscle actin (SMA) double immunohistochemistry was performed on 109 BC tissue specimens from patients aged 35 to 79 years old, followed by microscopic evaluation of cellular and vascular components inside peritumor adipose tissue. Microscopic findings were then correlated to age, body mass index (BMI), lymphovascular (LVI) and perineural invasion (PnI), recurrence (R), and tertiary lymphoid structures (TLSs). **Results**: Four BCAAT subtypes have been identified as fibroblast-rich (F^Rich^_BCAAT), myofibroblast-rich (MyoF^Rich^_BCAAT), vascular-rich (V^Rich^_BCAAT), and mixed-vascular and inflammatory-rich (VI^Rich^_BCAAT). The F^Rich^_BCAAT subtype predominates for the age subgroup 35 to 49 years old and is a significantly worse prognostic factor for survival (*p* = 0.022). For the age subgroup of 50 to 69 years old, the VI^Rich^_BCAAT subtype significantly influences PnI (*p* = 0.05) but not survival (Log-rank test, *z* = 0.57, *p* = 0.57). V^Rich^_BCAAT was significantly impactful for BC patient survival aged 70 to 75 years old (*p* = 0.043). BMI did not correlate with any of the BCAAT subtypes but was strongly correlated with prognostic markers for each BCAAT subtype. **Conclusions**: Based on immunohistochemically detected cellular and vascular components, four microscopic BCAAT subtypes were identified. Three of four BCCAT subtypes specifically affect BC patient prognosis and survival depending on age.

## Simple Summary

Breast cancer-associated adipose tissue (BCAAT) is a well-established subject within the field. However, the data concerning its influence on prognosis and survival continues to evoke considerable debate, likely stemming from an incomplete characterization of the phenomenon. Our objective was to highlight the BCAAT subtypes microscopic heterogeneity through an immunohistochemical evaluation of cellular and vascular elements while also examining their implications for prognosis and survival outcomes. The present paper defines four subtypes of BCAAT, each exhibiting distinct overlaps across various age subgroups. Furthermore, these subtypes demonstrate a statistically significant influence on invasion and recurrence independent of body mass index (BMI) or the presence of immune-related microscopic structures such as tertiary lymphoid structures (TLSs). Future investigations will be essential to determine whether BCAAT subtypes may influence therapeutic responses and resistance.

## 1. Introduction

The term “tumor-associated adipose tissue” or “breast cancer-associated adipose tissue” (BCAAT) refers to the adipose tissue that is located around a breast tumor and undergoes alterations and interactions with the cancer [1]. Through the secretion of a variety of chemokines, growth factors, enzymes, and free fatty acids, cancer-associated adipocytes (CAAs) acquire a more “aggressive” nature, which promotes the growth of tumors, the spread of metastases, and resistance to treatment [2,3,4]. Performing research on breast CAA tissue, often known as BCAAT, is an area that is both exciting and complex. It is not only a passive energy reserve. Rather, it is dynamically involved in the development of breast cancer (BC), as well as in its spread and therapy resistance [2,5]. There is a myriad of epithelial cancers that either develop close to or spread to fat cells [6]. When cancer cells communicate with adipose tissue, it alters adipocyte function and paracrine signaling, creating an environment favorable for tumor growth [2]. Recent years have shown that tumor cells transform adipocytes in the tumor microenvironment (TME) into CAAs or cancer-associated adipocytes [2]. Bidirectional signaling fosters a metabolic symbiosis between adipocytes and cancer cells when they engage [5,7,8]. It is possible for tumor cells to “re-educate” normal adipocytes in breast tissue to transform into CAAs [2]. They undergo morphological, gene expression, and secretory profile alterations because of this transition [4,9].

The importance of BCAAT in BC development and invasion is not restricted to the local invasion but also to the ability of the BC cell to spread and to induce distant metastasis [10,11,12]. Occasionally, distant metastatic processes are adipose tissue-dependent, as is the case for BC bone metastasis [12]. Tumor cells significantly disrupt the strictly regulated cellular and molecular connections inside the bone marrow, promoting their own survival and proliferation [10]. The function of bone-resorbing osteoclasts in BC bone metastases is well recognized. However, the roles of other bone cells, adipocytes, endothelial cells, and nerve cells remain poorly understood [12]. Leptin and adiponectin, secreted by adipose cells, are the two most prominent adipokines and have some effects on how cancer cells behave that are opposite to each other [13]. Adiponectin plays a role in glucose absorption and fatty acid catabolism [13]. Adiponectin has an anti-tumor effect on the skeleton by encouraging apoptosis and slowing the growth of BC cells [13]. This is accomplished by the stimulation of many pathways including mTOR and NF-κB signaling in BC cells [13]. Sadly, this anticancer effect is less effective in people who are overweight because they have less adiponectin receptors, which makes them resistant to treatment [13]. Some investigations have controversially demonstrated that adiponectin can promote BC migration and proliferation [14,15]. Leptin, on the other hand, promotes tumors and is linked to higher rates of metastasis [13,16]. For instance, leptin and IL-1β from adipocytes promote the migration and colonization of BC cells in the adipose tissue of the skeleton [10]. Leptin plays a role in controlling VEGF in BC cells through hypoxia-inducible factor 1α (HIF-1α) and NF-κB signaling, which encourages cancer growth [13].

With age, the bone marrow undergoes progressive replacement of its hematopoietic parenchyma with adipose tissue, and sentinel lymph nodes may also exhibit fatty infiltration and structural remodeling [17]. Recent data suggested that axillary lymph nodes can also be affected, especially in obese women [17]. Some authors previously reported that a significant positive correlation between larger axillary lymph nodes and axillary metastases in obese individuals diagnosed with BC was detected [13]. Although fatty nodes are a benign variety compared to metastatic nodes, their findings indicate that larger fat-expanded axillary lymph nodes may serve as an imaging indicator for axillary metastases in obese women [17]. A morphologic analysis of fat-enlarged axillary lymph nodes recently performed by Song et al. [18] demonstrated an augmented average adipocyte size in nonmetastatic lymph nodes of node-positive individuals. The same authors demonstrated by immunohistochemistry (IHC) a diminished CD3 expression and an elevated leptin expression in the fat-infiltrated axillary lymph nodes of obese node-positive patients. The findings indicate a new way for exploring the relationship between lymph node, obesity, lymphatic dysfunction, and BC nodal metastases, emphasizing a potential predictive instrument for obese BC patients [18]. Obesity is one of the main factors inducing extracellular matrix remodeling, which subsequently affects treatment response and resistance [9]. Despite wide research on BCAAT at the molecular level, its morphology and histopathology are not extensively reported in the literature [4]. Highly heterogeneous BCAAT morphology identified by a histopathologist and certified by IHC may become one of the prognostic tools for pathologists at initial diagnosis.

BCAAT heterogeneity usually refers to the basic morphology of white, brown, pink, and beige adipose tissue, as well as to the content of fat vacuoles released into the cytoplasm [9]. Some studies refer to changes in the size and shape of BCAAT cells adjacent to the tumor [19] while completely neglecting other adipose tissue components that may be present within BCAAT near tumor areas, such as inflammatory cells, fibroblast and myofibroblast content, and adipose tissue vascularity. Most of the BCAAT studies are focused on the triple-negative BC subtype (TNBC) [20], with data for other BC subtypes being very limited.

Recently published papers have continued to confer a high importance to adipose tissue related to its influence on BC development, invasion, metastasis, but also to prognosis and survival [21,22,23,24]. Tong et al. [22] defined a cellular atlas of adipocyte heterogeneity across molecular subtypes of BC, shedding light on the subtype-specific contributions of adipocytes to the modification of the TME.

Given the reasons described above, we aim to characterize BCAAT at the invasive front and in areas close to malignant regions, including additional BCAAT components beyond adipose tissue cells, such as blood vessels, fibroblasts, and myofibroblasts within the adipose tissue and inflammatory cells. After classifying BCAAT morphologically and immunohistochemically based on the tissular components mentioned above, we will study the impact of each BCAAT subgroup on patient survival rate, TNM staging parameters, and other clinicopathologic and microscopic parameters.

## 2. Materials and Methods

### 2.1. Ethical Considerations in Selecting Patients and Related Selection Criteria

Participating in this study is a group of 109 women admitted to the OncoHelp Clinic (https://oncohelp.ro accessed on 12 December 2026) Timisoara, Romania, ranging in age from 35 to 79 years old, who were diagnosed with ductal invasive carcinoma through histological evaluation between 2015 and 2022. The tissues were preserved by the formalin-fixed paraffin-embedded (FFPE) technique. To validate the diagnosis and determine which cases were suitable for IHC, two independent pathologists further examined the FFPE samples. An IHC marker-based molecular profile was generated for every patient. Hormone receptors (ER, PR), proliferative index (Ki67), and human epidermal growth factor receptor 2 (HER2) assessments were among the markers. All reagents were kindly provided by Leica Biosystems, Deer Park, IL, USA. The various molecular subtypes of BC could not be better understood without this. Fifty-three out of 109 BC patients had a complete clinicopathologic profile with clinical, histological, and therapeutic criteria useful for this investigation. Important clinicopathologic and therapeutic variables are analyzed in this study, including age, BMI, lymphovascular invasion, perineural invasion, recurrence, and survival. The Research Ethics Council of the Victor Babeș University of Medicine and Pharmacy in Timișoara, Romania (No. 49/28.09.2018) investigated and authorized the use of a standardized form to acquire informed consent from every patient. Also, it is important to mention that due to the low number of final selected cases, this is an exploratory analysis.

The inclusion criteria we followed were: age over 18, patients with a certified diagnosis of BC on the initial biopsy, no diabetes history or other metabolic diseases, no history of significant cardiovascular disease which may affect adipose tissue by edema, no history of autoimmune disease or other inflammatory conditions, a complete pathologic profile of BC (TNM staging, grade, IHC surrogate markers for molecular classification, neoadjuvant and adjuvant therapy protocol), calculation of the body mass index (BMI), TLS presence, premenopausal or postmenopausal status, the informed consent obtained from the BC patients, and their positive response about the use of their biopsies for research.

Age below 18 years old, lack of informed consent, presence of diabetes and other metabolic diseases, autoimmune disease, and other inflammatory conditions, cardiovascular disease and renal failure certified before or during BC diagnosis were considered the exclusion criteria.

### 2.2. Objectives

The main objective of the current study is to classify BCAAT according to cellular and vascular components. An additional objective includes the correlation of BCAAT subtypes with clinicopathologic and prognostic parameters. We quantified local inflammatory cells organized as tertiary lymphoid structures (TLSs), CD34+ fibroblasts and SMA-positive myofibroblasts, but also CD34+ blood vessels, all part of BCAAT adjacent to the malignant areas.

### 2.3. Basic Procedures, Histological Examination, and Selection Criteria for Formalin-Fixed Paraffin-Embedded Specimens for Immunohistochemical Evaluation

Tumor samples were obtained by needle core biopsy for initial diagnosis confirmation. The tumor and its surrounding tissue were selected for testing as a representative sample. The BC tissue samples were prepared according to standard protocol, which included obtaining the tissues, storing them in buffered formalin for 24 to 48 hrs and then embedding them in paraffin. The FFPE block was meticulously cut into three micrometer-thick portions and then those sections were delicately placed onto glass slides. Hematoxylin and eosin were used to stain a slide that was obtained from each case for histopathologic analysis. To confirm the initial histological diagnosis and assess the quality of the tissue, two independent pathologists examined the relevant hematoxylin- and eosin-stained slides.

### 2.4. Immunohistochemistry

To assess the overall quality of the tissue, immunostaining with vimentin (clone V9) was employed. Patients’ slides where positive vimentin staining was detected in the tumor stroma were chosen for further IHC testing. Vimentin staining is an easy-to-use IHC test to validate if the tissue quality is proper for IHC [25]. Patient slides where positive vimentin staining was detected in the tumor stroma were chosen for further IHC testing.

Using an auto-stainer made by Leica Biosystems, (Newcastle upon Tyne, UK), three-micrometer-thick slices were subjected to IHC. The unmasking approach made use of the Novocastra Bond Epitope Retrieval Solutions 1 and 2 made by Leica Biosystems. For 5 min, endogenous peroxidase activity was inhibited using a 3% hydrogen peroxide solution. In order to investigate whether BC cells exhibited the SMA+ response, a double immunostaining strategy was used on several tissue samples. A thorough examination of the distinct characteristics of developed and undeveloped tumor blood vessels coming from the stromal component of BC has been conducted and assessed before. For 30 min at room temperature, the endothelium of the tumor vasculature was targeted using CD34 mouse anti-human monoclonal antibodies (clone QB) (Leica Biosystems). Furthermore, we incubated the samples at room temperature for 30 min using mouse SMA anti-human monoclonal antibodies (clone 1A4) (Leica Biosystems). Visualization systems like the Bond Polymer Refine Red Detection System ( Leica Biosystems, Deer Park, IL, USA) and the Bond Polymer Refine Detection System DAB (Leica Biosystems, Deer Park, IL, USA) were necessary and used as the next step of the IHC staining. The endothelium of the vessels was used as a positive control for CD34 staining and to identify blood vessels inside adipose tissue adjacent and far from malignant areas. Positive controls for SMA staining were perivascular cells surrounding stromal capillaries [26]. Skipping incubation with the primary antibody yielded the negative control. We evaluated the SMA cytoplasmic expression in stromal myofibroblasts to identify their presence within the adipose tissue adjacent to malignant areas, and CD34 expression to identify blood vessels located within the same peritumoral adipose tissue compartment.

### 2.5. Digital Image Analysis

Following scanning with an OCUS 20 Microscope (Grundium, Tampere, Finland), the IHC samples were saved as SVS files in the Case Center Slide Library (3DHistech, Budapest, Hungary). All slides stained with CD34 and SMA or CD34 alone were imported into QuPath version 0.4.3, an open-source digital pathology platform for bioimage analysis [27]. An accurate evaluation of tumor stromal blood vessels was made possible by utilizing the integrated software as Fiji (https://imagej.net/software/fiji (accessed on 13 January 2026) ImageJ distribution, Laboratory for Optical and Computational Instrumentation, University of Wisconsin, Madison, WI, USA) and Vascular Analysis (QuPath extension module, Queen’s University Belfast, Belfast, UK) to examine the slides. In brief, we used the brush tool to construct three to five region of interests (ROIs) within the cancerous areas. This allowed us to delineate the tumor regions containing SMA tumor cells with the highest degree of accuracy. Determination of stain vectors was part of the pre-processing phase that started the digital image analysis (DIA). After that, we configured cell and intensity parameters and selected the positive cell detection option to start cell identification. To exclude the nucleus, the detection picture was calibrated to estimate the total optical density using a pixel size of 0.5 μm and a cell expansion of 1.988 μm. There was a score compartment and three evaluation levels for the intensity threshold parameters: low (+1, in yellow), medium (+2, in orange), and high (+3, in red). The QuPath application allowed an automated cell count and the percentage of BC cells that were SMA-positive or SMA-negative. A combined Stromal Score (SS) that combines the density and intensity of positively detected cells was also available in addition to the usual density and intensity scores. This was reminiscent of the Allred score, a semi-quantitative IHC scoring system that combines the proportion of SMA-positive tumor cells and staining intensity to generate a composite score ranging from 0 to 8 [28]. A histological score (H-score) was also a part of the QuPath analysis. To provide a more precise assessment and correlation with clinicopathologic parameters, this study employed the intensity, density, and Allred Score of BC cells positive for SMA.

### 2.6. Network Formation Assay

Adipose tissue morphology heterogeneity in normal and malignant breast tissue was assessed by using the Network Formation Assay (NFA) application from the IKOSA platform (Kolaido, St. Gallen, Switzerland), adapted for tissue assessment. This application highlighted morphology heterogeneity and differences between NBAAT and BCAAT with a higher accuracy by the assessment of branching points, loops, and cell coverage.

### 2.7. Statistical Data Analysis

Applying JAMOVI (Available online: https://www.jamovi.org (accessed on 12 January 2026). The Jamovi Project, Sydney, Australia) on macOS systems allowed for statistical analysis. Age, BMI, menopausal status, lymphovascular (LVI) and perineural invasion (PnI) were all factors linked to the DIA results. We performed a survival rate analysis by using the Kaplan–Meier survival test. If the *p*-value was less than 0.05, we considered that there was a statistically significant correlation.

## 3. Results

### 3.1. Normal Breast-Associated Adipose Tissue Characterization and BC-Associated Adipose Tissue Microscopic Subtypes

**Figure 1:**
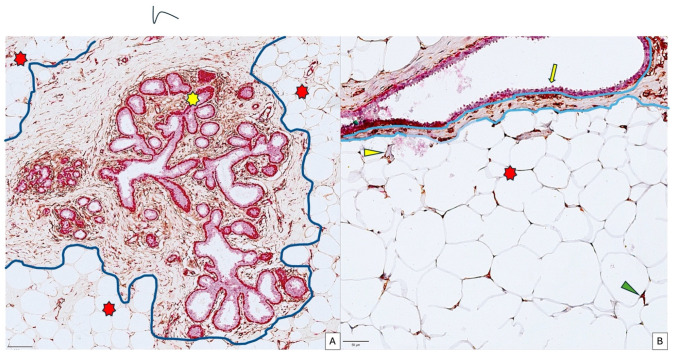
Adipose tissue from the normal breast (NBAAT) surrounds secretory and ductal components of the normal mammary gland. NBAAT (indicated by the red star in both images) is separated by normal breast epithelial components by a variable amount of dense, irregular connective tissue rich in CD34+ fibroblasts, which protect TDLU ((**A**), yellow star) and lactiferous ducts ((**B**), scale bar 50 μm), encircled by the blue lines) from direct contact with NBAAT. Together with large polygonal white adipose cells, rare CD34-positive fibroblasts ((**B**), green arrowhead), and tiny scattered capillaries ((**B**), yellow arrowhead) exemplify the microscopic image of NBAAT. For BCAAT, the dense, irregular connective tissue interposed between malignant areas and adipose tissue is completely lacking in cases with tumor cells invading the surrounding adipose tissue, and, when this connective tissue persists, it has reduced thickness and presents a myofibroblastic reaction rich in SMA-positive cells .

Normal breast-associated adipose tissue (NBAAT) has been detected around the terminal ductal lobular unit (TDLU) from normal breast tissue adjacent to tumor areas (Figure 1A,B). No direct contact between TDLU components and surrounding adipose tissue has been observed. For all cases, a variable area of dense, irregular connective tissue has been interposed between TDLU, the main ducts, and the NBAAT. Regarding the NBAAT histology, the adipose tissue has a lobulated appearance composed of white adipose cells. The histology of normal breast adipose tissue revealed large adipocytes, which have a nucleus on the periphery and a transparent, unique lipid droplet in the middle. A stroma rich in fine collagen fibers supports these adipocytes, which also comprise extremely rare other cell types, such as fibroblasts, highlighted by CD34 immunolabelling.

**Figure 2:**
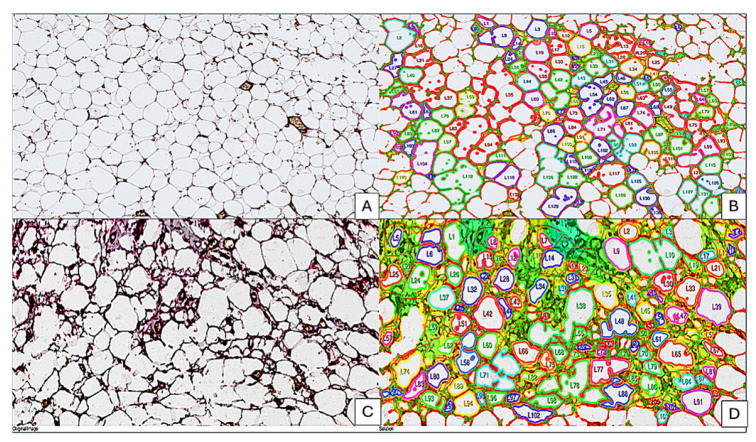
Comparative assessment of the NBAAT (**A**,**B**) and BCAAT (**C**,**D**) architecture. In NBAAT, white adipose cells are orderly arranged and intermingle with a few small capillaries (**A**). For BCAAT, a chaotic morphology has been observed with the presence of multivacuolated adipose cells compared with normal adipose cells, which have a unique lipid vacuole (see panel (**A**)). This modified architecture with a changed adipose tissue cell morphology was certified by NFA assessment, where the number of loops and branching points was significantly higher for BCAAT (**D**) compared to NBAAT (**B**).

Together with large polygonal white adipose cells, rare CD34+ fibroblasts (B, green arrowhead), and tiny scattered capillaries (B, yellow arrowhead) exemplify the microscopic image of NBAAT. For BCAAT, the dense, irregular connective tissue interposed between malignant areas and adipose tissue is completely lacking in cases with tumor cell invasion of the surrounding adipose tissue. When this connective tissue persists, it has reduced thickness and presents a myofibroblastic reaction rich in SMA-positive cells. (Figure 2A,B).

BCAAT had several morphologic differences related to white adipose cells, but a high heterogeneity related to other connective tissue cell subtypes. By applying the IKOSA NFA test, we found that BCAAT is composed of smaller white adipose cells, highly heterogeneous in shape, size, and morphology and closely packed and invaded by a well-developed vascular network of small capillaries (Figure 2C,D).

**Figure 3:**
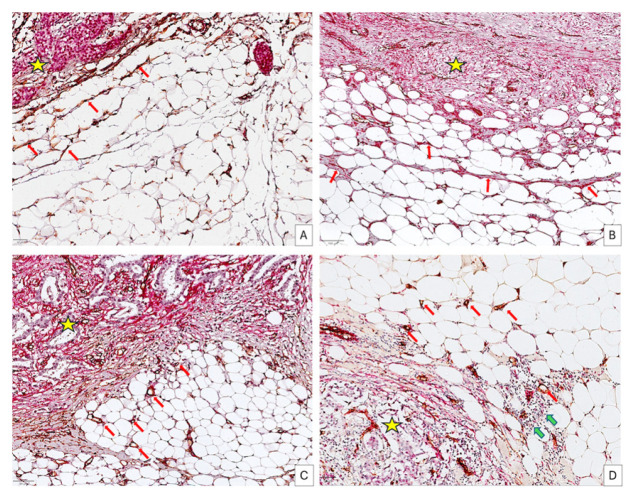
BCAAT subtypes adjacent to BC malignant areas (yellow star) according to their cellular and vascular components. The fibroblast-rich subtype (**A**) has no blood vessels but is rich in CD34+ fibroblasts ((**A**), red arrows, scale bar 50 μm). The myofibroblast-rich subtype is devoid of vasculature but contains SMA-positive myofibroblasts embedded within adipose cells, but also in high numbers in connective tissue trabeculae ((**B**), red arrows, scale bar 100 μm). For the vascular-rich subtype (**C**), scale bar 50 μm), a high density of small blood vessels of the capillary type is observed inside the adipose tissue ((**C**), red arrows). For the mixed vascular inflammatory subtype, a mixture of blood vessels ((**D**), red arrows) and inflammatory cells (**D**), green arrows, scale bar 50 μm) was observed.

Four different BCAAT subtypes have been defined according to cellular and vascular components, (1) fibroblast-rich, (2) myofibroblast-rich, (3) vascular-rich, and (4) mixed-vascular and inflammatory-rich (Figure 3).

All these types refer to the adipose tissue immediately adjacent to malignant areas and were classified according to the morphology of the adipose tissue cells, the presence of fibroblasts and myofibroblasts embedded within the adipose cells, the presence of blood vessels, and the diffuse infiltrate of inflammatory cells. For the fibroblast-rich BCAAT (F^Rich^_BCAAT), adipose cell morphology is similar to NBAAT but a high number of CD34+ fibroblasts are inserted in between them. The density of CD34+ fibroblasts gradually decreases from the tumor vicinity to the adipose tissue periphery (Figure 3A). No evidence of an increased capillary density compared to NBAAT has been observed within the F^Rich^_BCAAT. Myofibroblast-rich BCAAT (MyoF^Rich^_BCAAT) is composed of adipose tissue cells heterogeneous in size and shape, showing a discrete lobulation given by the presence of the connective tissue trabecula rich in SMA-positive myofibroblasts (Figure 3B). Also, for this subtype, the vascular network is similar to NBAAT, and no diffuse inflammatory infiltrate has been observed. The vascular-rich BCAAT subtype (V^Rich^_BCAAT) is composed of small adipose cells, some of them with features of beige adipose tissue. But the most prominent component of this subtype is a vascular network composed of a high density of small capillaries with an obvious lumen predominantly distributed close to the malignant areas (Figure 3C).

The mixed vascular inflammatory-rich subtype BCAAT (VI^Rich^_BCAAT) has diffused inflammatory cells intermixed with a well-developed capillary network and adipose cells. Moreover, inflammatory cells were distributed in a crown-like fashion around most of the adipose cells (Figure 3D).

### 3.2. BCAAT Subtypes Distribution According to Clinicopathologic Parameters

We stratified the patients according to age and BCAAT subtypes. Analysis of BCAAT subtypes according to age revealed that VI^Rich^_BCAAT subtypes are the most predominant for the age group of 35 to 49 and 50 to 69 years old, while V^Rich^_BCAAT was found as the most common subtype for the age group between 70 and 79 years old (Table 1).

**Table 1:** BCAAT subtypes stratification according to patient age. BCAAT, breast cancer-associated adipose tissue; FRich, fibroblast-rich; MyoFRich, myofibroblast-rich; VIRich, vascular inflammatory-rich; VRich, vascular-rich.

Age (Years) Number of Patients	35–49 (n = 18)	50–69 (n = 17)	70–79 (n = 18)
BCAAT (%)			
MyoF^Rich^	5.26	11.53	11.11
F^Rich^	26.31	23.07	11.11
V^Rich^	26.31	23.07	44.5
VI^Rich^	42.10	42.30	33.33

The next step in the age-related evaluation of BCAAT subtypes was to determine if different subtypes may influence other clinicopathologic parameters. For the 35 to 49 age subgroup, BCAAT subtypes were significantly positively correlated to months of survival (*p* = 0.022) and negatively correlated to lymphovascular invasion LVI (*p* = −0.016) and to R (*p* = −0.004) (Table 2).

**Figure 4:**
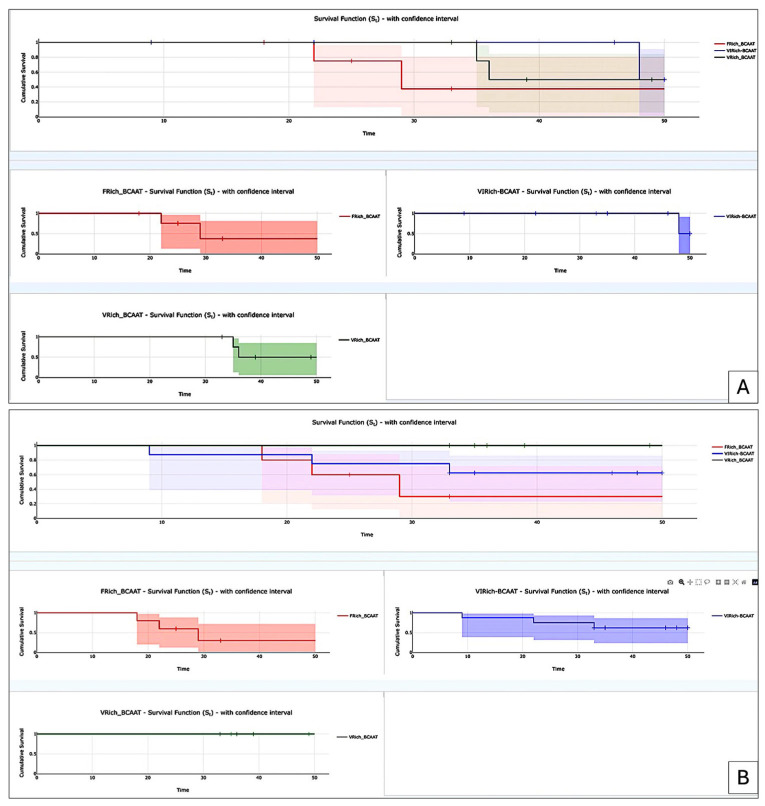

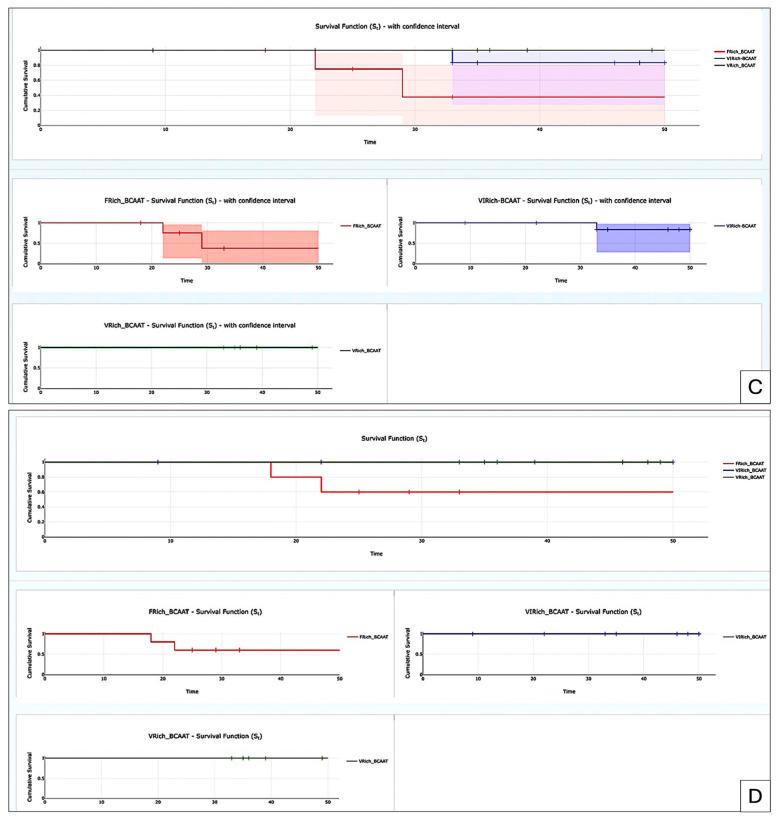
Kaplan–Meier survival curves were assessed according to BCAAT subtypes in relation to death event (**A**), as well as to the presence of LVI (**B**), PnI (**C**), and R (**D**).

It has been noted that survival was correlated both to BCAAT subtypes and to BMI. No significant impact of BCAAT subtypes has been identified on PnI but a significant impact has been found for LVI and R. The Kaplan–Meier survival test showed differences related to overall survival between different types of BCAATs. As shown in Figure 4A, the lowest cumulative survival rate was found for the F^Rich^_BCAAT subgroup. When we analyzed BCAAT subgroups related to the presence of lymphovascular invasion, we also observed that F^Rich^_BCAAT subgroup had the lowest survival rate compared to other subgroups, even though the decrease in survival appeared earlier for the VI^Rich^_BCAAT subgroup. Similar results were observed for both Pnl and R related to FRich_BCAAT. Based on these results, we may assume that the F^Rich^_BCAAT subgroup may have a significant impact on the prognosis and survival of BC patients from the 35 to 49-year-old group.

**Figure 5:**
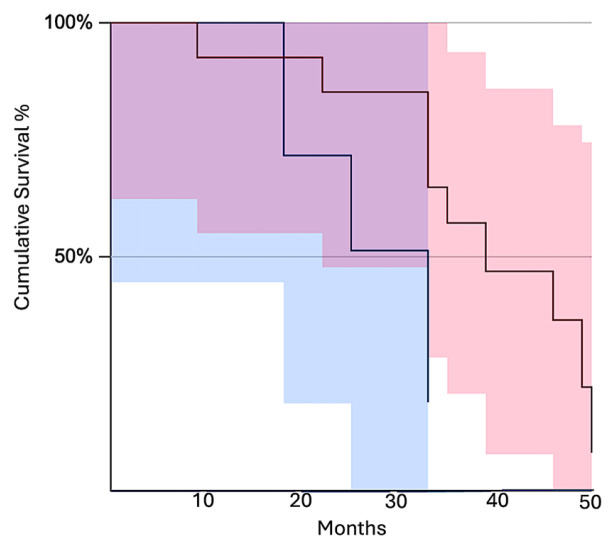
Kaplan–Meier survival analysis between vascular and fibroblastic subgroups for the age subgroup from 35 to 49 years old. The fibroblast and myofibroblast subgroups had a lower survival rate (blue line) compared to the vascular subgroup (red line). The red and blue shaded areas represent the 95% confidence intervals of the survival curves.

After analysis of BCAAT subtypes related to clinicopathologic parameters, we assessed the impact of a high vascular network on patient survival rate. To this end, we compared BCAAT subtypes rich in blood vessels to the group rich in fibroblasts and myofibroblasts. Survival rates were significantly different between the vascular subgroup and the fibroblast and myofibroblast subgroups (*z* = 2.02, *p* = 0.043) (Figure 5).

**Figure 6:**
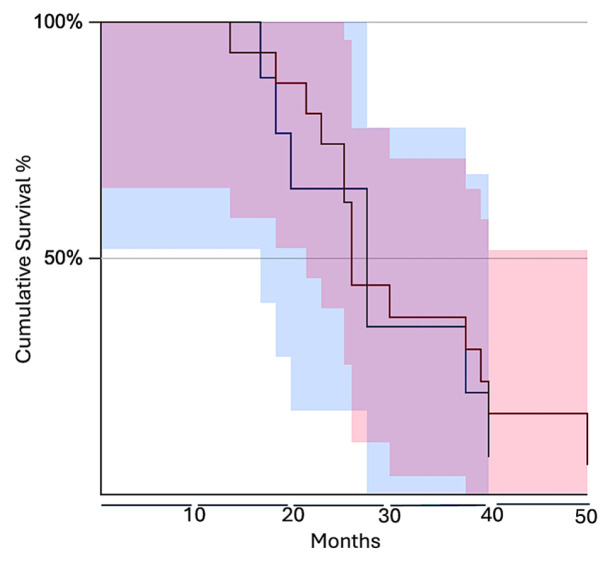
Kaplan–Meier survival analysis between vascular and fibroblastic subgroups for the age subgroup from 50 to 69 years old. The fibroblast and myofibroblast subgroups had a lower survival rate (blue line) compared to the vascular subgroup (red line). The red and blue shaded areas indicate the 95% confidence intervals corresponding to each survival curve.

The overall BCAAT subtypes analysis for the age group of 50 to 69 years old failed to yield any significant correlations between BCAAT subtypes and clinicopathological parameters included in the study. The only significant correlation was detected between the presence of TLSs and BMI (*p* = 0.033), unrelated to any BCAAT subtype. Also, the presence of TLSs seems to negatively influence PnI (*p* = −0.019). When we separately analyzed the vascular subgroups (V^Rich^_BCAAT and VI^Rich^_BCAAT), we found a significant inverse correlation between BCAAT subtypes and age (*p* = 0.029). V^Rich^_BCAAT was predominant for the age up to 55 years old while VI^Rich^_BCAAT was predominantly found for ages over 60 years old. For PnI, a significant positive correlation was found for VI^Rich^_BCAAT (*p* = 0.05). The presence of TLSs was significantly influenced by BMI (*p* = 0.005). The survival analysis for this subgroup, by comparing fibroblast and myofibroblast versus vascular subgroups, showed no significant differences between these two groups (log-rank test, *z* = 0.57, *p* = 0.57, Figure 6).

The most common BCAAT subtypes found for the group aged between 70 and 75 were VI^Rich^_BCAAT and V^Rich^_BCAAT with a total percentage of 77.83% out of the total cases. Global analysis for both VI^Rich^_BCAAT and V^Rich^_BCAAT for this age group revealed no significant differences between these adipose tissue subtypes. When we separately analyzed the vascular BCAAT subgroup, we found a significant correlation between months of survival and V^Rich^_BCAAT (*p* = 0.043) but also between BMI and LVI (*p* < 0.001), BMI and PnI (*p* < 0.001), and R and both LVI (*p* = 0.03) and PnI (*p* = 0.03) (Table 3).

## 4. Discussion

One of the most common and dangerous illnesses that impacts women globally is still BC. The malignant epithelial cells that propel tumor growth have historically been the focus of investigation. Recent research, however, emphasizes how important the TME—especially adipose tissue—is in accelerating the spread of cancer [6]. Adipose tissue, which makes up a substantial portion of the breast’s unique composition, may have a direct role in the development, spread, and metastasis of BC according to new research [1,25,29].

Human breast adipose tissue represents a dynamic stromal compartment that actively participates in metabolic regulation, paracrine signaling, and structural support of the mammary gland [30]. Breast adipocytes are interspersed within connective tissue, fibroblasts, blood vessels, and immune cells [19]. Age, hormone levels, and body composition all affect the ratio of adipose to glandular (milk-producing) tissue [30,31]. Recent studies stated that BCAAT is different compared to NBAAT [19,30,31].

The first aspect observed as being different between NCAAT and BCAAT was the morphology of the adipose cells, and thus most of the research on the adipose tissue from the normal and malignant breast was focused on the changes found in the morphology of the adipose cells [4,9,30,31,32,33] during malignant progression. Immunophenotyping of other cellular and vascular components, except macrophages, was studied less or was completely neglected. The present study classified BCAAT based not only on the morphology of adipose cells but also on quantifying the presence of fibroblasts, myofibroblasts, and inflammatory cells together with vascular components. We identified four BCAAT subtypes: (1) fibroblast-rich, (2) myofibroblast-rich, (3) vascular-rich, and (4) mixed-vascular and inflammatory-rich. Wolf et al. [4] partially used similar criteria to highlight differences between NBAAT and BCAAT, but their study did not define BCAAT subtypes and was solely restricted to TNBCs. Also, they comparatively assessed cellular differences in adipose tissue for normal-weight and obese women with TNBC exclusively. We included in our study all BC molecular subtypes. We did not perform a comparative analysis of the impact on all four BCAAT subtypes because we focused on defining the four types of BCAAT and on first identifying whether they may have an impact on clinicopathologic parameters. The comparative evaluation of BCAAT between BC molecular subtypes will be the aim of one of our next efforts.

Based on CD34 IHC, we were able to define a fibroblast-rich BCAAT subgroup (F^Rich^_BCAAT) where peritumor adipose tissue contains a high density of CD34+ cells. CD34+ fibroblasts exert a protective role in normal breast stroma but usually decrease within the tumor-associated stromal dense irregular connective tissue [26,34], whereas their role inside peritumoral adipose tissue remains controversial. In BC, CD34+ cells located within the adjacent adipose tissue constitute a diverse group of progenitor and stromal cells that play a significant role in facilitating tumor progression and metastasis [35,36]. CD34 is a protein marker for various types of progenitor cells, including hematopoietic stem cells, endothelial cells, and adipose-derived stem and stromal cells (ADSCs) [37]. The dual function of CD34 expression is crucial for diagnosis and treatment. CD34 is a prevalent marker utilized to identify cancer stem cells in some malignancies, which are linked to heightened tumor aggressiveness [6,38,39]. We found here that F^Rich^_BCAAT is specific for women up to 50 years old and strongly influences LVI, PnI, and R for this age subgroup. Based on these observations, we demonstrated that the F^Rich^_BCAAT subgroup had the lowest survival rate compared to other BCAAT subgroups for women with BC aged between 35 and 49 years old. CD34+ adipose cells were previously mentioned as a negative prognostic factor in BC, mostly in the experimental models [40,41,42] and to a lesser extent in human BC specimens, but this phenomenon was not related to age, nor to LVI, PnI, or recurrence, in contrast to the present study. Our results show that F^Rich^_BCAAT has a clinical impact on the prognosis and survival of non-menopausal BC patients, most probably due to an endocrine-related mechanism. This hypothesis is sustained by previously published studies. In a work by Hamel et al. [43], a comparative analysis was conducted to identify differences between young and aged ER-α+ breast tumors based on the observation that younger patients with estrogen receptor-alpha-positive (ER-α+) BC are more resistant to endocrine treatments, suggesting the activation of alternative estrogen signaling pathways. They found a matrix and paracrine enrichment factor in tumor samples from younger patients (≤40 years old), as opposed to older patients (≥65 years old). The ability of the tumor stroma to modify estrogen signaling was assessed by analyzing adipose-derived stromal and stem cells (ASCs) from noncancerous lipoaspirate of young and elderly donors for changes in matrix synthesis and paracrine secreted substances. While ASCs of different ages showed similar levels of proliferation, differentiation, and matrix formation, they differed in the expression of inflammatory cytokines, including interleukin IL-2,-6,-8 and -10, interferon gamma, and tumor necrosis factor alpha. Experiments utilizing conditioned media revealed that the younger age of adipose-derived stem cell donors enhances the endocrine response in ER-α+ BC cell lines. The MCF-7 ER-α+ BC cell lines, when exposed to secreted factors from young adipose-derived stem cells, exhibited an upregulation of ER-α-regulated genes, such as PGR and SDF-1, in contrast to MCF-7 cells treated with conditioned medium from aged adipose-derived stem cells [43]. This previous indirect experimental evidence strongly supports our findings on human tissues. Despite their heterogeneous nature, in the present study, we used the term “fibroblasts” due to their morphology. Several data from the literature mention that BC tumor cells induce lipolysis into BCAAT and support adipose cells dedifferentiation into CD34+ fibroblasts or SMA-positive myofibroblasts [44]. Research indicates that adipocytes subjected to factors secreted by cancer cells undergo a process of dedifferentiation, resulting in the loss of their lipid droplets and a transformation into fibroblasts [2,5]. The altered cells exhibit traits akin to cancer-associated fibroblasts (CAFs), which are dynamic stromal cells that engage in the remodeling of the extracellular matrix and release factors that promote tumorigenesis [2,5]. The transition from adipocyte to fibroblast plays a significant role in the tumor stroma, exhibiting heightened rigidity, augmented mobility and penetration of cancer cells and persistent inflammation within the TME [1,13]. We assessed CD34-positive fibroblasts within BCAAT and found that this fibroblast-rich subtype is specific to a particular age group, namely non-menopausal women, and strongly influences prognosis and survival compared to the other BCAAT subtypes described here.

**Figure 7:**
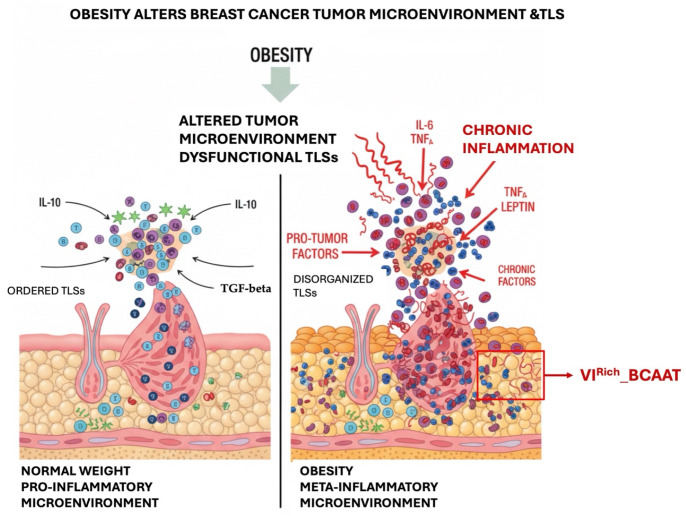
Schematic representation of the dual role of TLSs in overweight and obese BC patients according to age and BCAAT subtype. In patients up to 55 years old with the vascular-rich BCAAT (VRich_BCAAT) subtype, TLS presence is associated with a pro-inflammatory microenvironment that correlates inversely with PnI. In contrast, in patients older than 60 years with the vascular-inflammatory-rich BCAAT (VIRich_BCAAT) subtype, TLS presence is associated with a meta-inflammatory microenvironment that correlates positively with PnI and worse prognosis. The schematic highlights the age-dependent and microenvironment-dependent behavior of TLS within distinct BCAAT subtypes.

For the age group between 50 and 69 years, no significant differences were detected between BCAAT subgroups. However, we observed a significant association between BMI and the presence of TLSs. Overweight and obese patients seem to have more TLSs in the tumor stroma compared to normal-weight patients. In patients with obesity and BC, TLSs within the TME play a significant role. These structures are often abundant in the context of chronic inflammation associated with excess adipose tissue and may serve as sites of local anti-tumor immune responses [45]. However, they are also associated with poorer outcomes in certain scenarios, which may impede the effectiveness of immunotherapy, despite their general association with favorable prognoses [45]. This underscores a complex interplay where inflammation induced by obesity alters the TME and the function of TLSs, positioning them as targets for novel therapeutic approaches [45,46,47,48]. Our findings suggest that overweight and obese patients with BC exhibit increased TLS production due to a pro-inflammatory microenvironment, whereas the association between TLS presence and PnI appears to vary according to age and BCAAT vascular subtypes. Based on our findings, in patients up to 55 years old with the VRich_BCAAT subtype, the presence of TLS appears to be associated with a pro-inflammatory microenvironment that may protect against PnI, as suggested by the identified significant inverse correlation. In contrast, the VIRich_BCAAT subtype appears to be associated with a meta-inflammatory microenvironment in patients older than 60 years that favors PnI and is linked to worse prognosis. A schematic drawing illustrating the dual role of TLSs in overweight and obese BC patients is presented in Figure 7.

The clinical and therapeutic impact of these two different states is differentially summarized in Table 4.

Recent data are in line with our findings regarding a particular inflammatory microenvironment given by the presence of adipose tissue for postmenopausal patients with BC [49]. Because obesity-induced inflammation has been linked to an increased risk of BC in postmenopausal women with higher BMI, researchers are paying special attention to this phenomenon [24]. Obesity-induced inflammation causes proinflammatory cytokines, adipokines, immune cells, and tumor cells to infiltrate the increased adipose tissue of postmenopausal women with BC, changing the tumor’s immunologically driven milieu [6]. In the present study, we microscopically defined the VI^Rich^_BCAAT subtype and we found that BMI is significantly correlated with LVI and PnI. Compared to the BC subgroup aged from 50 to 69 years old, we found that V^Rich^_BCAAT but not VI^Rich^_BCAAT showed a significant impact on survival for the postmenopausal subgroup aged from 70 to 75 years. A high BCAAT vascularity is still controversial regarding its impact on BC patients’ survival, invasion, and metastasis. It is well accepted that BCAAT secretes a high amount of VEGF, which, in turn, induces an accelerated angiogenesis in both the tumor and stromal area by forming immature leaky neo-vessels, facilitating tumor cell spread locally or at a distance from the tumor. Low-dose metronomic chemotherapy “normalize” both tumor and stromal blood vessels, including those from BCAAT [50,51,52]. Chemotherapy aids in restoring the compromised architecture of tumor vasculature, hence reducing hyperpermeability. Chemotherapy enhances blood flow by eliminating immature capillaries and facilitating the maturation of the remaining ones, resulting in enhanced functional vascular density. Enhanced perfusion mitigates tumor hypoxia, therefore, diminishing the secretion of angiogenic factors such as VEGF that promote aberrant vessel formation. Low-dose chemotherapy elevates TSP-1 (thrombospondin-1) levels, functioning as anti-angiogenic agents, resulting in vascular normalization instead of destruction. In the current study, we found an increased survival for patients aged 70 to 75 years, where the V^Rich^_BCAAT subtype predominated, compared to the age subgroup 35 to 49 years old, which is governed by F^Rich^_BCAAT with low or no adipose tissue hypervascularization and a low survival rate. This difference could be explained by vascular normalization induced by chemotherapy not only in tumor tissue but also in peritumor adipose tissue. Changes in fatty tissue play a big role in postmenopausal BC, since fat turns into the main source of estrogen through the enzyme aromatase. More body fat, especially abdominal fat, causes inflammation and factors that help tumors grow, which raises the risk of hormone-receptor-positive BC. With age and especially through menopause, breast-associated adipose tissue is in continuous remodeling related to adipose cell changes and to vascular network changes. In BC, the adipose tissue-associated vascular network shows a similar change to tumor vascularization related to increased angiogenesis and the presence of a high density of small leaky immature blood vessels. Neoformation blood vessels favor lymphovascular invasion and tumor cell dissemination, and this may explain our findings related to the significant correlation between the V^Rich^_BCAAT subtype, LVI and PnI for the postmenopausal group. On the other hand, the neoadjuvant and adjuvant therapy received by our patients may induce vascular normalization, which in turn increases the effectiveness of chemotherapy drugs locally. This may explain the significant correlation between months of survival and the V^Rich^_BCAAT subtype for BC patients aged 70 to 75 years old. On the contrary, for patients aged 35 to 49 years, lack of vessels in the F^Rich^_BCAAT subtype reduces chemotherapy penetrance around the tumor and facilitates local spread, but it seems to exert a protective role on LVI and PnI. The fibroblast-rich microenvironment of the F^Rich^_BCAAT subtype induces fibrosis, facilitating local invasion. This may explain the low survival rate for premenopausal BC patients with F^Rich^_BCAAT subtype.

Also, BMI was found to be significantly impactful on LVI and PnI, and R seems to be partially related to LVI for this subgroup. Recently, Fuller et al. [53] reported an increased vascularity of the adipose tissue adjacent to cancer areas and that for the BC patients with a higher BMI, the vascular density of the adipose tissue is significantly higher compared to normal-weight patients. The authors did not stratify these findings according to age, as we demonstrated. Moreover, by using deep learning-based semantic segmentation, Whitmarsh et al. [27] demonstrated that in breast tissue close to malignancy, a greater vascular density was seen in adipose-rich tissue as opposed to stroma-rich tissue. The vascular density, which is the number of blood vessels per square millimeter in the adipose region stroma, is substantially higher (*p* < 0.05) than the vascular density in the rest of the stroma. This implies that greater vascularization in the stroma is linked to the proximity to adipose tissue. Furthermore, they demonstrated that the stromal vasculature in the vicinity of the tumor is both smaller and denser than the vasculature located farther from the tumor. These results further support the widely held belief that neo-vascularization occurs close to the tumor [50,51]. Our findings partially overlap with these previous observations, and moreover, we proved the strong prognostic impact of V^Rich^_BCAAT for the old BC patients related to LVI and PnI and linked them with recurrence.

The main strengths of the current study are (1) the first-time description of four different microscopic subtypes of BCAAT based on cellular and vascular components; (2) BCAAT subtypes analysis based on age subgroups, highlighting that their heterogeneity and subtype predominance are age dependent; (3) certification of BCAAT subtypes with different impacts on invasion and survival. The limited sample size of the present study may be mitigated by the continuous inclusion of additional cases. The lack of detailed correlation between BCAAT and BC molecular subtypes represents another limitation

The data will be published in a future paper. Future directions related to BCAAT subtypes are to find if these four subtypes are distinct subtypes or different evolutionary steps related to other factors, such as nutrition, occupational behaviors or mental stress.

A balanced eating pattern with a high intake of unrefined grains, fruits, vegetables, nuts, and olive oil and a moderate to low intake of red meat and saturated fatty acids may increase overall survival following a BC diagnosis, according to data from the published literature. Patients’ quality of life is negatively impacted by a range of symptoms experienced by BC patients receiving chemotherapy and/or radiation. Nutritional counseling and supplementation with certain dietary elements, such as EPA and/or DHA, may be helpful in reducing drug-induced adverse effects and improving therapeutic efficacy, according to studies looking into nutritional interventions during BC treatment. As a result, dietary management may be regarded as a crucial component of the multimodal therapy strategy for BC patients. To increase long-term survival and quality of life, more research employing dietary therapies in extensive clinical trials is necessary to conclusively identify successful interventions in these patients [28]. Recently, Popescu et al. [54] highlighted that antineoplastic treatment should be complemented by suitable nutrition to optimize antitumor effectiveness and enhance the patient’s quality of life based on in vitro and in vivo experimental models and assumed that these studies need validation in a large cohort of patients in clinical trials. This special diet could have an impact on reshaping BCAAT, but at the moment, the data are very scarce.

The findings of this study may not be able to be generalized to a wider population yet due to several limitations, such as the small sample size, the retrospective design, and the dependence on data from a single center. In addition, we did not link our findings with the molecular subtypes of BC since the paper might become too long and this may impede its understanding and readability. This may have a temporary negative impact on the biological significance of our findings, but we are already preparing our next paper, which includes the assessment of BCAAT subtypes according to BC molecular subtypes and neoadjuvant and adjuvant therapy. In addition, there is a possibility that the results were affected by biases in the assessment of digital image analysis (DIA) and in the selection of participants due to the inclusion criteria for the cohort. In subsequent research, the primary objectives should be to validate these findings in bigger, multi-center cohorts, integrate analyses with genetic BC subtypes, and conduct prospective studies to further prove therapeutic value and minimize bias.

Tumor stage, nodal status, receptor status, and adjuvant therapy are common criteria widely used to assess survival. There is a lot of information about their impact on BC patients’ survival (29,480 papers for tumor stage impact, 2191 for nodal status, 35,976 for receptor status and 17,239 for adjuvant therapy) and thus, we considered that, correlated to our aim, they might have a minor impact. By contrast, only 284 papers were reported related to peritumor adipose tissue impact on survival (with a lot of controversies in between results), and none of them analyze the BCAAT microscopic subtypes’ impact on survival.

## 5. Conclusions

Within the scope of this investigation, four distinct microscopic subtypes of BCAATs were identified. These subtypes were determined by the IHC detection of cellular and vascular components. Three of the four subtypes of BCCAT have a considerable impact on prognosis and survival, and this impact is closely connected with age subgroups. Another subtype of BCCAT does not have any significant impact. The influence of FRich_BCAAT on survival was found to be significant for premenopausal women who had BC, even though vascular subtypes were found to be influential for women who had reached menopause. On the other hand, the influence of vascular subtypes differed depending on the age subgroups of women who had gone through menopause. Future investigation will refine and complete the BCAAT subtypes, as well as investigate the influence that these subtypes have on therapy and other prognostic markers.

## Figures and Tables

**Table 2:** Correlation between BCAAT subtypes and clinicopathologic parameters for the age group of 35 to 49-year-old. BCAAT, breast cancer-associated adipose tissue; BMI, body mass index; LVI, lymphovascular invasion; PnI, perineural invasion; R, recurrence. * *p* < 0.05; ** *p* < 0.01; *** *p* < 0.001.

		BCAAT Subtypes		Survival (Months)	
Survival (months)	Pearson’s r	0.522	*	—	
	*p*-value	0.022		—	
	Spearman’s rho	0.613	**	—	
	*p*-value	0.005		—	
	Kendall’s Tau B	0.489	**	—	
	*p*-value	0.010		—	
BMI	Pearson’s r	0.143		0.526	*
	*p*-value	0.559		0.021	
	Spearman’s rho	0.084		0.443	
	*p*-value	0.731		0.058	
	Kendall’s Tau B	0.088		0.378	
	*p*-value	0.677		0.053	
LVI	Pearson’s r	−0.546	*	−0.737	***
	*p*-value	0.016		<0.001	
	Spearman’s rho	−0.538	*	−0.771	***
	*p*-value	0.017		<0.001	
	Kendall’s Tau B	−0.502	*	−0.660	**
	*p*-value	0.022		0.001	
PnI	Pearson’s r	−0.285		−0.167	
	*p*-value	0.236		0.495	
	Spearman’s rho	−0.321		−0.239	
	*p*-value	0.180		0.325	
	Kendall’s Tau B	−0.299		−0.204	
	*p*-value	0.173		0.312	
R	Pearson’s r	−0.624	**	−0.506	*
	*p*-value	0.004		0.027	
	Spearman’s rho	−0.586	**	−0.543	*
	*p*-value	0.008		0.016	
	Kendall’s Tau B	−0.547	*	−0.465	*
	*p*-value	0.013		0.021	

**Table 3:** Correlation matrix for the V^Rich^_BCAAT, BMI, LVI, and PnI for patients in the 70 to 75 age range with BC. * *p* < 0.05; ** *p* < 0.01; *** *p* < 0.001. BMI, body mass index; CI, confidence interval; LVI, lymphovascular invasion; PnI, perineural invasion.

		V^Rich^_BCAAT		Survival (Months)	BMI		LVI		PnI	
Survival (months)	Pearson’s r	0.568	*	—						
	*p*-value	0.043		—						
	95% CI upper	1.000		—						
	95% CI lower	0.022		—						
	Spearman’s rho	0.500		—						
	*p*-value	0.071		—						
	Kendall’s Tau b	0.443		—						
	*p*-value	0.067		—						
BMI	Pearson’s r	−0.375		−0.693	—					
	*p*-value	0.857		0.987	—					
	95% CI upper	1.000		1.000	—					
	95% CI lower	−0.768		−0.901	—					
	Spearman’s rho	−0.378		−0.305	—					
	*p*-value	0.859		0.804	—					
	Kendall’s Tau b	−0.365		−0.261	—					
	*p*-value	0.872		0.819	—					
LVI	Pearson’s r	−0.356		−0.414	0.893	***	—			
	*p*-value	0.844		0.883	<0.001		—			
	95% CI upper	1.000		1.000	1.000		—			
	95% CI lower	−0.759		−0.787	0.671		—			
	Spearman’s rho	−0.356		−0.144	0.943	***	—			
	*p*-value	0.844		0.654	<0.001		—			
	Kendall’s Tau b	−0.356		−0.128	0.910	**	—			
	*p*-value	0.857		0.667	0.002		—			
PnI	Pearson’s r	−0.356		−0.414	0.893	***	1.000	***	—	
	*p*-value	0.844		0.883	<0.001		<0.001		—	
	95% CI upper	1.000		1.000	1.000		1.000		—	
	95% CI lower	−0.759		−0.787	0.671		1.000		—	
	Spearman’s rho	−0.356		−0.144	0.943	***	1.000	***	—	
	*p*-value	0.844		0.654	<0.001		<0.001		—	
	Kendall’s Tau b	−0.356		−0.128	0.910	**	1.000	**	—	
	*p*-value	0.857		0.667	0.002		0.001		—	
R	Pearson’s r	0.327		0.178	0.547		0.612	*	0.612	*
	*p*-value	0.178		0.311	0.051		0.030		0.030	
	95% CI upper	1.000		1.000	1.000		1.000		1.000	
	95% CI lower	−0.275		−0.415	−0.008		0.091		0.091	
	Spearman’s rho	0.327		0.529	0.577	*	0.612	*	0.612	*
	*p*-value	0.178		0.058	0.040		0.030		0.030	
	Kendall’s Tau b	0.327		0.469	0.557	*	0.612	*	0.612	*
	*p*-value	0.163		0.056	0.042		0.033		0.033	

**Table 4:** Clinical, prognostic, and therapeutic impact of TLS presence related to BMI [45,46,47,48].

Aspect	Lean Patients	Overweight/Obese Patients
TLS formation	More frequent, well-organized	Less frequent or disorganized
Immune profile	Pro-inflammatory (anti-tumor)	Meta-inflammatory (pro-tumor)
Prognosis	Improved survival with TLS	TLS may not confer same benefit
Therapy response	Better response to immunotherapy and/or chemotherapy	May need adjusted or combination immunotherapies

## Data Availability

Data are unavailable due to privacy or ethical restrictions and can be requested from A.M.C.

## Abbreviations

BC	breast cancer
BCAAT	breast cancer-associated adipose tissue
BMI	body mass index
CAA	cancer-associated adipose tissue
CAAs	cancer-associated adipocytes
CD3	cluster of differentiation 3
DAB	3,3′-diaminobenzidine
DIA	digital image analysis
ER	estrogen receptor
ER-α	estrogen receptor alpha
IKOSA	artificial intelligence-based image analysis platform
IL-1β	interleukin-1 beta
LVI	lymphovascular invasion
mTOR	mammalian target of rapamycin
NBAAT	normal breast-associated adipose tissue
NF-κB	nuclear factor kappa B
PnI	perineural invasion
PR	progesterone receptor
PGR	progesterone receptor gene
R	recurrence
ROIs	regions of interest
SDF-1	stromal cell-derived factor 1
SMA	smooth muscle actin
SVS	scanned virtual slide format
TLSs	tertiary lymphoid structures
TNM	tumor-node-metastasis
VEGF	vascular endothelial growth factor
